# The Notch Pathway Is Important in Maintaining the Cancer Stem Cell Population in Pancreatic Cancer

**DOI:** 10.1371/journal.pone.0091983

**Published:** 2014-03-19

**Authors:** Ethan V. Abel, Edward J. Kim, Jingjiang Wu, Mark Hynes, Filip Bednar, Erica Proctor, Lidong Wang, Michele L. Dziubinski, Diane M. Simeone

**Affiliations:** 1 Departments of Surgery, University of Michigan, Ann Arbor, Michigan, United States of America; 2 Department of Internal Medicine, University of Michigan, Ann Arbor, Michigan, United States of America; 3 Department Molecular and Integrative Physiology, University of Michigan, Ann Arbor, Michigan, United States of America; 4 Department Translational Oncology Program, University of Michigan, Ann Arbor, Michigan, United States of America; Schulze Center for Novel Therapeutics, Mayo Clinic, United States of America

## Abstract

**Background:**

Pancreatic cancer stem cells (CSCs) represent a small subpopulation of pancreatic cancer cells that have the capacity to initiate and propagate tumor formation. However, the mechanisms by which pancreatic CSCs are maintained are not well understood or characterized.

**Methods:**

Expression of Notch receptors, ligands, and Notch signaling target genes was quantitated in the CSC and non-CSC populations from 8 primary human pancreatic xenografts. A gamma secretase inhibitor (GSI) that inhibits the Notch pathway and a shRNA targeting the Notch target gene Hes1 were used to assess the role of the Notch pathway in CSC population maintenance and pancreatic tumor growth.

**Results:**

Notch pathway components were found to be upregulated in pancreatic CSCs. Inhibition of the Notch pathway using either a gamma secretase inhibitor or Hes1 shRNA in pancreatic cancer cells reduced the percentage of CSCs and tumorsphere formation. Conversely, activation of the Notch pathway with an exogenous Notch peptide ligand increased the percentage of CSCs as well as tumorsphere formation. In vivo treatment of orthotopic pancreatic tumors in NOD/SCID mice with GSI blocked tumor growth and reduced the CSC population.

**Conclusion:**

The Notch signaling pathway is important in maintaining the pancreatic CSC population and is a potential therapeutic target in pancreatic cancer.

## Introduction

Pancreatic cancer is the fourth most common cause of cancer-related death in the United States despite being the 10^th^ most common cancer diagnosis [1]. The high mortality rate is partly due to the fact that the vast majority of pancreatic cancers are diagnosed at an advanced stage. But at least equally important is that pancreatic cancers are generally only minimally responsive to chemotherapy and radiotherapy. There is increasing evidence that this resistance to therapy is at least in part due to the inherent resistance of a subpopulation of cancer cells that are tumorigenic and share many properties with stem cells and thus have been labeled cancer stem cells (CSC). Cancer stem cells were first isolated in myeloid leukemia [2] and were shown to share stem cell properties such as potential for self-renewal and the ability to differentiate and proliferate. Subsequently, CSCs have also been identified in a wide range of solid tumors including breast, brain, liver, colon, prostate, melanoma, and pancreatic tumors [3]–[10]_ENREF_3. Pancreatic cancer stem cells (CSC) were first isolated from human pancreatic cancers using the marker profile ESA^+^/CD44^+^/CD24^+^ [10]. These marker positive CSCs were able to form tumors in NOD/SCID mice that appeared histologically similar to the primary tumor, suggesting multi-potency with reconstitution of the various tumor cell types. In vitro evidence for a stem cell phenotype such as self-renewal was demonstrated by the ability to form tumor spheres *in vitro* in contrast to ESA^-^/CD44^-^/CD24^-^ cells which could not.

It remains incompletely understood how pancreatic cancer stem cells are maintained in a heterogeneous tumor. One potential contributor to CSC maintenance is the Notch signaling pathway. In the normal developing pancreas, the Notch signaling pathway has been shown to be an important regulator of the balance between self-renewal and differentiation [11]–[13]. There are 4 members of the mammalian Notch receptor family (NOTCH 1–4) which are similarly processed and activated through a series of cleavage events. The mature Notch receptor is composed of two subunits that are generated as a result of an initial cleavage event by furin-like convertase [14]. Notch signaling pathway activation occurs when a Notch receptor binds to one of the five known Notch ligands [jagged1 (JAG1), jagged2 (JAG2), delta-like 1 (DLL1), delta-like 3 (DLL3), and delta-like 4 (DLL4)]. Ligand binding causes a conformational change in the Notch receptor which allows a second cleavage by tumor necrosis factor-alpha-converting enzyme (TACE) [15]. A third cleavage event is carried out by a gamma secretase (presenilin), which releases the intracellular domain of Notch allowing it to translocate to the nucleus to activate expression of target genes [16].

Inhibition of the Notch signaling pathway results in depletion of multi-potent pancreatic progenitor cells [11], [13]. Conversely, induced Notch activation prevents pancreatic epithelial differentiation and results in increased maintenance of undifferentiated pancreatic progenitor cells [12]. Based on similar phenotypic characteristics exhibited by CSCs, the Notch signaling pathway has been evaluated for its role in CSC self-renewal. Both breast and brain CSCs have been shown to have increased Notch pathway activation [17], [18]. In vitro inhibition of the Notch signaling pathway in these two tumor types results in decreased self-renewal, shown by reduction in tumorsphere formation. We hypothesized that the Notch signaling pathway is further upregulated in pancreatic CSC and contributes to pancreatic CSC function and pancreatic cancer tumorigenesis.

In this study, we evaluated the role of the Notch pathway in maintaining the CSC population and its effects of inhibition in pancreatic tumor growth. We detect upregulation of several Notch pathway components in pancreatic CSCs and demonstrate that inhibition by a gamma secretase inhibitor or shRNA to Hes1, a key Notch target gene, reduces pancreatic CSC self-renewal and tumorigenicity. In vivo treatment of established orthotopic pancreatic tumors with a gamma secretase inhibitor reduces tumor growth and combination with cytotoxic chemotherapy further augments the anti-tumor response. Our results suggest that Notch signaling is critical for pancreatic CSC maintenance and that targeting the Notch signaling pathway in pancreatic cancer has promising therapeutic potential.

## Materials and Methods

### Ethics Statement

Pancreatic adenocarcinoma tissue samples were obtained from patients that underwent surgical procedures within the University of Michigan Health System. Written informed consent from all research subjects was obtained prior to collection of tissue, and all protocols involving patient samples were reviewed and approved by the Institutional Review Boards of the University of Michigan Medical School (IRBMED). Original, hardcopies of all written informed consent forms are kept within a secure file at the University of Michigan. The Institutional Review Boards of the University of Michigan Medical School (IRBMED) determined that this study conforms to applicable guidelines, state and federal regulations, and the University of Michigan's Federalwide Assurance (FWA) with the Department of Health and Human Services (HHS). The IRBMED also approved all written consent documents, as well as consent procedures, for this study. The University of Michigan Federalwide Assurance number for this study is FWA00004969, and the Study University of Michigan study identification number is HUM00025339.

### Antibodies and reagents

Merck gamma secretase inhibitor (MK-0752) was provided by Dr. Max Wicha (University of Michigan) and was used for the *in vitro* and *ex vivo* studies. Gamma secretase inhibitor RO4929097 was kindly provided by Roche (Indianapolis, IN) and was used for the *in vivo* studies. PE-conjugated mouse anti-human CD44 and FITC-conjugated mouse anti-human CD24 antibodies were purchased from B.D. (Franklin Lakes, NJ). APC conjugated mouse anti-human ESA was purchased from Miltenyi Biotech and biotinylated mouse anti-mouse H2K was purchased from Southern Biotech. Hes1 antibody used for Western Blot was obtained from Dr. Xing Fan (University of Michigan) or purchased from MBL International (Woburn, MA). Notch1 and cleaved Notch1 antibodies were purchased from Cell Signaling Technology (Danvers, MA) and β-actin antibody was purchased from Sigma (St. Louis, MO). Matrigel was purchased from B.D. (Franklin Lakes, NJ). Hes1 shRNA clone V2LHS 249784 was purchased from Open Biosystems (Huntsville, AL). The delta/Serrate/Lag-2 (DSL) peptide (sequence CDDYYYGFGCNKFCRPR) was synthesized by the University of Michigan Protein Structure Facility.

### Protocol approval

Animal protocols were approved by University Committee for the Use and Care of Animals (UCUCA) at University of Michigan and lentivirus protocols were approved by Institutional Biosafety Committee (IBC) at University of Michigan.

### Preparation of single cell suspensions of tumor cells

Single cell suspension of tumor cells was prepared as described [10] with the following modifications. Primary human pancreatic adenocarcinoma xenograft tissue was minced completely and then suspended in 200 Unit/mL ultrapure collagenase IV (Worthington Biochemicals, Freehold, NJ) in Media 199 (Invitrogen, Carlsbad, CA). After enzyme digestion at 37°C for 45 to 60 minutes and mechanical dissociation by pipetting every 15 minutes with a 10 mL pipette, the digested and dissociated cells were filtered through a 40 μm nylon mesh cell restrictor (B.D. Franklin Lakes, NJ) and washed with HBSS (Invitrogen, Carlsbad, CA) twice. The cells were then resuspended in 2% FBS in HBSS for experiments.

### Flow cytometry

Flow cytometry was performed as described previously [10]. Dissociated cells were counted and transferred to 5 mL tubes, washed with HBSS/2%FBS twice and resuspended in HBSS/2% FBS at a concentration of 1 million cells per 100 μL. Sandoglobin (1 mg/mL) was added to the sample at a dilution of 1∶50 and the sample was incubated on ice for 20 min, then washed twice with HBSS/2%FBS. Antibodies PE-conjugated mouse anti-human CD44, FITC-conjugated mouse anti-human CD24, APC conjugated mouse anti- human ESA and biotinylated mouse anti-mouse H2K were added at the dilution as instructed, and the sample was incubated for 45 min on ice and then washed twice with HBSS/2%FBS. Streptavidin conjugated with APC-Cy7 was added at the dilution of 1∶200 and the sample was incubated on ice for 15 minutes. After washing twice with HBSS/2%FBS, cells were resuspended in HBSS/2%FBS containing 3 μM 4′,6-diamidino-2-phenylindole (DAPI) (Invitrogen, Carlsbad, CA). Flow cytometry was done using a FACSAria (B.D., Franklin Lakes, NJ). Side scatter and forward scatter profiles were used to eliminate cell doublets.

### Quantitative PCR

Primers for Notch pathway components were selected from a primer bank (Wang & Seed, 2003) and synthesized by Invitrogen (Carlsbad, CA). Total RNA was extracted from sorted cancer stem cells and non-cancer stem cells using a RNeasy Micro kit (Qiagen, Valencia, CA) as instructed. Reverse transcription of cDNA was performed using High-Capacity cDNA Reverse Transcription Kits (Applied Biosystems, Foster City, CA) as instructed. qPCR was performed on Rotorgene 6000 (Qiagen, Valencia, CA) using a Power SYBR Green PCR Master Mix (Applied Biosystem, Foster City, CA) per the manufacturer's instructions, with all reactions normalized to GAPDH. Conditions used for qPCR were 95°C hold for 10 mins, 40 cycles of 95°C for 10 secs, 60°C for 15 secs, and 72°C for 20 secs.

### Tumorsphere cultures

Established pancreatic cancer tumorspheres [10] were maintained in sphere media as described previously [19], [20] with modifications [50% NeuralBasal (Invitrogen, Carlsbad, CA), 1% N2 Supplement (Invitrogen, Carlsbad, CA), 2% B27 supplement (Invitrogen, Carlsbad, CA), 1% Antibiotic-Antimycotic (Invitrogen, Carlsbad, CA), 10 ng/mL BMP4 (Sigma), 10 ng/mL LIF (Sigma), 20 ng/mL human bFGF-2 (Invitrogen, Carlsbad, CA), all in 1∶1 DMEM/F12 (Invitrogen, Carlsbad, CA)]. Tumorspheres were passaged every 6 days. For passaging, tumorspheres were dissociated with 0.05% trypsin for 2–5 min and then immediately washed twice with 40 mL PBS. Cells were then passed through a 40 μm nylon mesh cell strainer, counted and plated in fresh sphere medium in Costar ultra low-attachment 6 well plates (Corning, Lowell, MA).

### GSI treatment of tumorsphere cells

Tumorspheres were dissociated into single cells and re-suspended in sphere medium containing GSI at the indicated concentrations for the noted time periods. Cells were then observed under a microscope or harvested for analysis.

### Western blotting

Cells treated with or without GSI at various doses were lysed in Lysis buffer (20 mM Tris pH 7.5/150 mM NaCl/12 mM EDTA/10% glycerol/1% Triton X-100) containing a protease inhibitor cocktail (Roche Applied Science, Indianapolis, IN) and PhosphoStop (Roche Applied Science, Indianapolis, IN). The protein concentration was measured using a Bio-Rad Protein Assay (Bio-Rad, Hercules, CA). Fifty μg of protein was mixed with an equal volume of 2x SDS loading buffer (Invitrogen, Carlsbad, CA) and boiled for 5 min before applying the sample to SDS-PAGE. After SDS-PAGE, the protein gel was blotted on a nitrocellulose membrane Hybond C extra (Amersham Biosciences, Pittsburgh, PA) using a Bio-Rad blotting apparatus (BioRad, Hercules, CA) for one hour. The blot was blocked with 5% dry milk in TBST for one hour, washed twice in TBST, and then incubated with antibodies (1∶1000) in 5% BSA at 4°C overnight. The blot was washed three times in TBST and then incubated with peroxidase-conjugated secondary antibody (Jackson ImmunoResearch, West Grove, PA) in 5% dry milk at room temperature for 1 hour. After washing three times in TBST, the blot was incubated for 5 minutes with SuperSignal West Pico Chemiluminescent Substrate (Pierce, Rockford, IL) and X-ray film (Denville Scientific, Metuchen, NJ) was then developed.

### shRNA transductioin

Lentivirus constructs pGIPZ vector containing Hes1 shRNA or control shRNA were made in the Vector core at University of Michigan. Transduction was performed using a ViraDuctin Lentivirus Transduction Kit (Cell Biolabs, Inc. San Diego, CA) as instructed.

### Apoptosis assay

Tumorsphere cells transduced with control or Hes1 shRNA were cultured in sphere media containing 4 μg/mL puromycin for 4 days followed by trypsinization and PBS washing. Cells were filtered through a 40-μm nylon mesh to obtain single cell suspensions. The resultant single cells were fixed with ice cold 50% ethanol in PBS overnight. Cells were then stained with PI and FITC-annexin V using an Annexin V: FITC Apoptosis Detection Kit II purchased from BD Biosciences (San Jose, California).

### DSL treatment of tumorspheres

Spheres were dissociated into single cells and re-suspended in sphere medium containing DSL peptide at indicated concentrations for the indicated times. Treated cells were observed under microscope or harvested for analysis.

Ex vivo GSI treatment of tumorspheres and implantation into NOD/SCID mice

Single cells dissociated from tumorspheres were cultured in sphere medium containing DMSO or 8 μM GSI for 48 hours before being dissociated with trypsin and stained with DAPI and an antibody to H2K. Cells were sorted by FACS and DAPI negative and H2K negative cells were collected to obtain a pure live, human pancreatic cancer cell population. After washing with HBSS, sorted cells were re-suspended in sphere medium. Cell viability was validated using Trypan blue (Invitrogen, Carlsbad, CA). Forty thousand cells in 50 μL medium were mixed with an equal volume of Matrigel and injected subcutaneously into the midflank region of NOD/SCID mice using a 23 gauge needle. Five mice were injected for each treatment group. Tumor size was measured weekly. Tumors were not allowed to exceed 2 cm in diameter before euthanasia. Additionally, animals were monitored daily by the University of Michigan veterinary staff and sacrificed at any sign of distress or weight loss. At the end of the study animals were euthanized by carbon dioxide asphyxiation followed by cervical dislocation to ensure death.

### Orthotopic Implantation of Pancreatic Tumor Cells into NOD/SCID Mice and GSI Treatment

Pancreatic tumor cells incubated with luciferase-expressing lentivirus overnight were washed with serum free HBSS and suspended in a PBS/Matrigel mixture (1∶1 volume) at the concentration of 10 million cells/mL for implantation. Mice were anesthetized with an intraperitoneal injection of 100 mg/kg ketamine and 5 mg/kg xylazine, and a small left subcostal incision was performed. 500,000 bulk tumor cells in a volume of 50 μl were injected into the tail of the pancreas using a 30-gauge needle. Buprenorphine was administered every 6 hours for the first 24 hours post-surgery to alleviate pain. Treatment with chemotherapy was initiated 2 weeks after tumors were detectable. Three individual low passage human pancreatic xenograft tumors were included in the analysis. There were 4 groups of mice: control, RO4929097 (GSI), gemcitabine, and GSI plus gemcitabine, with 5 mice per group. Animals were evaluated by bioluminescent imaging and tumor growth was evaluated weekly. GSI (30 mg/kg) was administered by oral gavage at the frequency of 5 days on 2 days off. This schedule was used to minimize GSI-induced diarrhea secondary to goblet cell hyperplasia [21]. Gemcitabine (50 mg/kg) was delivered weekly by intraperitoneal injection as previously described [22]. Treatment was given for 4 weeks at which point animals were euthanized by carbon dioxide asphyxiation followed by cervical dislocation to ensure death. Prior to sacrifice, animals were monitored daily by the University of Michigan veterinary staff and sacrificed at any sign of distress or weight loss. Necropsy was performed and tumors were excised for analysis.

### Bioluminescent Imaging

Bioluminescent imaging of implanted orthotopic tumors in mice was performed using a Xenogen IVIS 200 Imaging System (Xenogen Biosciences, Cranbury, NJ) as previously described [23].

### Immunohistochemistry

Tissue samples were fixed in 10% phosphate-buffered formalin and embedded in paraffin. Formalin-fixed, paraffin-embedded sections were cut 4-μm thick, mounted on poly-l-lysine–coated slides (Sigma), and dried overnight at 37°C. Sections were then dewaxed in xylene, rehydrated according to standard histopathologic procedures, and stained with H&E. Immunodetection was done using the DakoCytomation LSAB+Kit according to manufacturer's protocol (Dako, Denmark). The slides were then counterstained with Hematoxylin and covered with VectaMount Mounting Media (Vector Labs, Burlingame, CA). Each stained section was then evaluated by microscopy.

### TUNEL Assay

TUNEL analysis was done using the Promega TUNEL assay kit (Promega, San Luis Obispo, CA) according to the manufacturer's instructions. Apoptotic cell nuclei were visualized as a yellow-green fluorescent signal under fluorescence microscopy. Cell nuclei were counterstained with DAPI (Vector Labs, Burlingame, CA).

*Additional reagents* RO4929097 for in vitro work was purchased from Selleck Chemicals (Houston, TX).

*Oligonucleotides for qRT-PCR* The following sequences were used: Hes1 5′-GAGAGGCGGCTAAGGTGTTTG-3′ and 5′-CTGGTGTAGACGGGGATGAC-3′, GLI1 5′-GGCTGGACCAGCTACATCAAC-3′ and 5′-TGGTACCGGTGTGGGACAA-3′, GLI2 5′-GCAAATGAAAGCCAGGGAAC-3′ and 5′-ATCTCAGGAAGGCGATGAAC-3′, PTCH1 5′-TATCCAGCACTTACTTTACGACCT-3′ and 5′-ATCCTGAAGTCCCTGAAGCC3′, and GAPDH 5′-TCACCAGGGCTGCTTTTAAC-3′ and 5′-GACAAGCTTCCCGTTCTCAG-3′.

### Statistical analysis

Data are expressed as the mean ± S.E. Statistically significant differences were determined by Student's *t* test and *Χ* ^2^ analyses or the Mann-Whitney U test where appropriate, and defined as *P*<0.05.

## Results

### Notch signaling pathway is upregulated in pancreatic cancer stem cells

**Figure 1:**
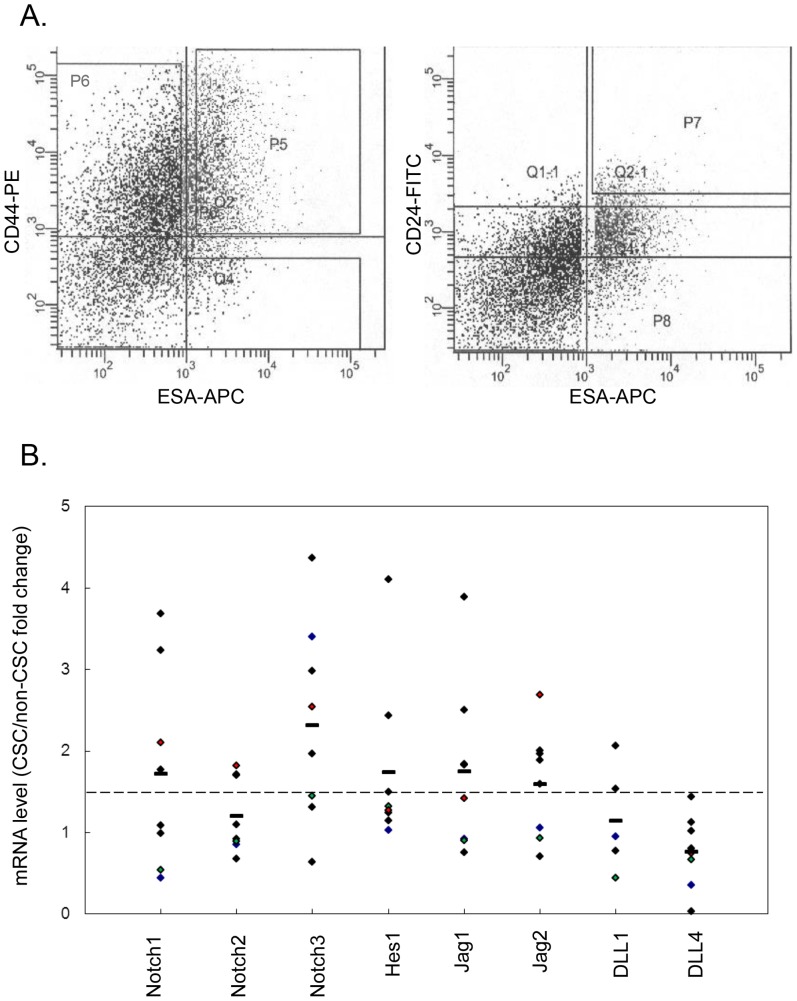
Expression profile of Notch pathway components in cancer stem cells (CSC). **A**. Flow cytometry analysis. Dissociated low-passage human pancreatic cancer xenograft cells were stained with DAPI and antibodies to H2K, ESA, CD44 and CD24. DAPI positive dead cells and H2K positive mouse cells were both eliminated from the analysis. The CSC population (with surface marker ESA^+^/CD44^+^/CD24^+^) was gated by both P5 and P7, and the non-CSC population from P6. **B**. Transcript levels of Notch pathway components in CSC compared to in non-CSC obtained by qPCR, normalized to GAPDH. The mean fold change between tumors is represented by a horizontal bar.

The Notch signaling pathway has been shown to be activated in pancreatic cancer cells [24]. We hypothesized that Notch signaling pathway components may be further upregulated in the pancreatic cancer stem cell subpopulation. Using primary human pancreatic cancer xenografts from 8 different patient tumors we evaluated expression levels of Notch signaling pathway components in cancer stem cells compared to bulk tumor cells. Tumors from xenografts were isolated and processed into single cell suspensions that were then sorted based on the triple-marker profile CD44+/CD24+/ESA+ to obtain CSCs and separately non-CSCs (Figure 1A). Quantitative RT-PCR analysis of RNA obtained from CSCs and non-CSCs for expression of components of the Notch signaling pathway was performed. The results suggest upregulation of Notch pathway components in CSCs above that of non-CSCs (Figure 1B). CSCs in 6 of 8 tumors expressed at least one Notch receptor at a level >1.5 fold over non-CSC. Similarly, at least one Notch ligand was upregulated >1.5 fold in CSC over non-CSC in 6 of 8 tumors. Of note, we did not detect expression of either the Notch4 receptor of DLL3 ligand in either CSC or non-CSC compartments in any of the primary xenografts tested (data not shown). There was a mean 1.75 fold greater expression of the Notch pathway target gene Hes1 in the CSCs compared to non-CSCs. These results suggest that CSCs differentially express increased levels of Notch pathway components and that the pathway is correspondingly activated as shown by enhanced expression of Hes1.

### Notch pathway inhibition

**Figure 2:**
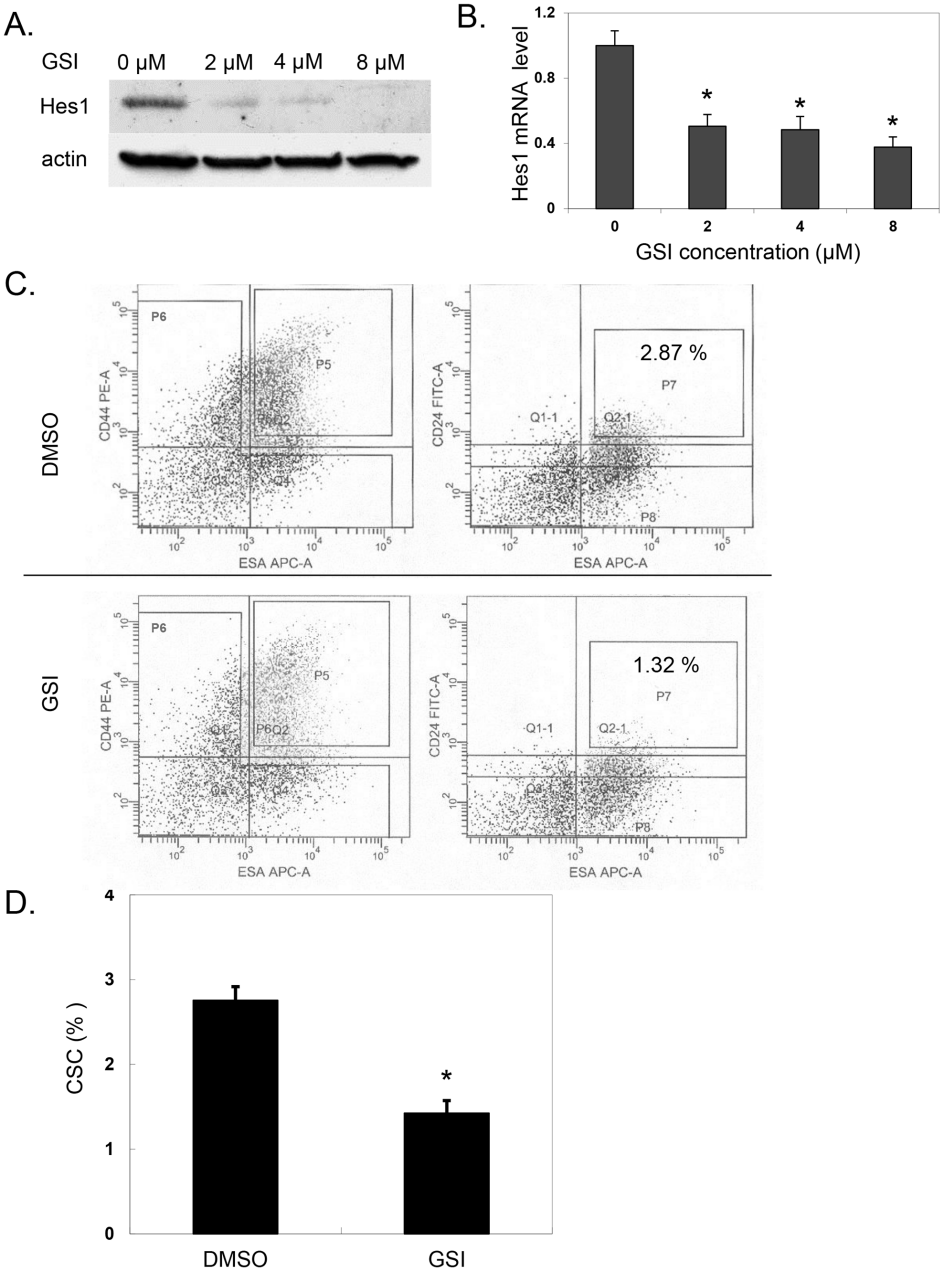
GSI treatment of pancreatic cancer cells. **A**. Primary pancreatic cancer cell tumorspheres (derived from patient 5) were cultured in sphere medium containing different concentration of GSI as indicated for 48 hours before Western blot analysis with an antibody to Hes1 or β-actin. **B**. Quantitation of Hes1 mRNA levels following GSI treatment for 48 hours (*p<.001 vs. control). **C**. Pancreatic cancer cells in sphere medium were treated with control vehicle (DMSO) or 8 μM GSI for 48 hours. FACS analysis of control DMSO treated cells (*upper panel*) and GSI treated cells (*lower panel*). CSC population with surface marker of ESA^+^/CD44^+^/CD24^+^ was identified by those cells in both gates P5 and P7. **D**. Quantitation of ESA^+^/CD44^+^/CD24^+^ CSC population following DMSO and GSI treatment (*p = 0.013 vs. DMSO).

Based on the observation that Notch signaling pathway components are upregulated in pancreatic cancer and in particular pancreatic CSC, we further studied the contribution of Notch pathway activation to pancreatic CSC function. Gamma secretase catalyzes the third cleavage step of the Notch receptor which releases the Notch intracellular domain. This step allows the Notch receptor to then translocate to the nucleus and activate Notch signaling, resulting in upregulation of target gene expression. Inhibition of gamma secretase with a gamma secretase inhibitor can thus block activation of the Notch signaling pathway. Incubation of pancreatic cancer tumorspheres with increasing doses of GSI reduced expression levels of the Notch target gene Hes1 in a dose dependent manner (Figure 2A,B) (p<.001 vs. control). Having confirmed that GSI can inhibit Notch signaling in pancreatic cancer cells, we next evaluated the effect of Notch pathway inhibition specifically on the CSC compartment. Pancreatic cancer cells treated with GSI showed a significant reduction in CSCs with the ESA^+^/CD44^+^/CD24^+^ marker profile compared to untreated cells (2.76±0.16% control vs. 1.43±0.15% with GSI p = 0.013) (Figure 2C,D). This result suggests a role for Notch pathway activation in CSC maintenance.

**Figure 3:**
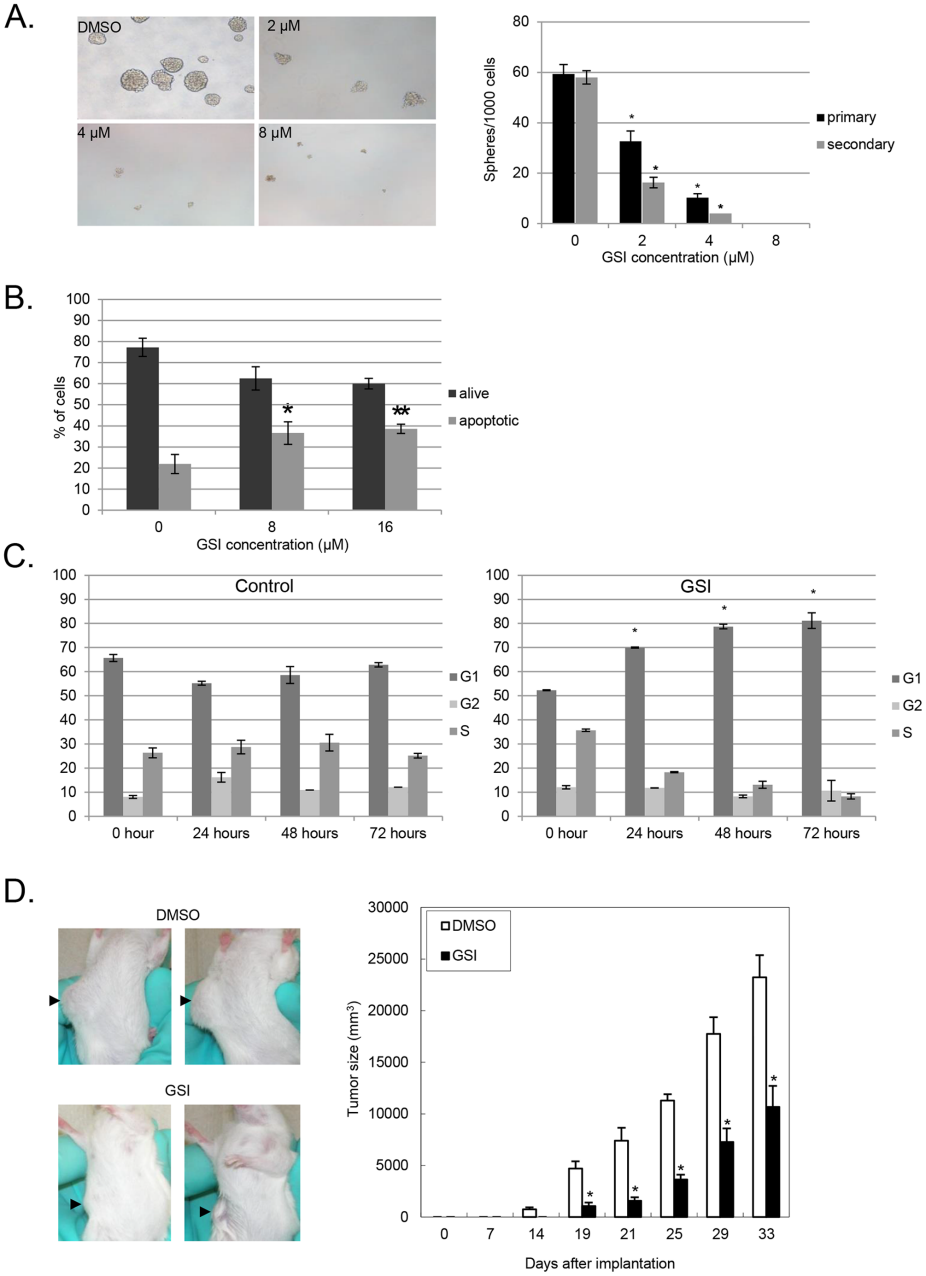
GSI treatment effect on pancreatic tumorspheres. **A**. Pancreatic cancer cell tumorspheres cultured for 5 days in sphere medium containing indicated concentrations of GSI. Representative images of primary tumorspheres that developed from 1000 cells per well cultured with increasing concentrations of GSI. Quantitation of number of primary tumorspheres (black bars) formed at each concentration with 1000 cells seeded per well (*p<.01 vs. control). Secondary tumorsphere formation (gray bars) from tumorspheres treated with GSI were dissociated into single cells, washed, and cultured for 5 days in normal sphere medium without GSI (**p<.01 vs. control). **B**. Tumorspheres treated with GSI at increasing concentrations stained with propidium iodide (PI) and FITC-Annexin V (AnV) and analyzed by flow cytometry to evaluate the extent of apoptosis.(*p<.05 8 μM vs. control, **p<.01 16 μM vs. control). **C**. Cell cycle analysis of pancreatic tumorspheres treated with DMSO control or GSI at 0, 24, 48, and 72 hrs. (*p<.01 vs. control). **D**. Pancreatic cancer cells cultured with either 8 μM GSI or vehicle control (DMSO) for 48 hours injected subcutaneously into NOD/SCID mice. Representative images of mice implanted with cells from the indicated treatment groups taken 21 days after injection. Arrows indicate location of the tumors. Serial tumor sizes measurements from mice treated with DMSO control or GSI at indicated time points. N = 5 (*p<.01 vs. control).

One of the defining features of CSCs is self-renewal which can be assayed in vitro by measuring tumorsphere formation through multiple passages. Pancreatic CSCs identified with the marker profile ESA^+^/CD44^+^/CD24^+^ have the ability to form spheres in nonadherent conditions which distinguishes them from non-cancer stem cells which lack this ability [10]. Having observed dose dependent inhibition of the Notch pathway with increasing doses of GSI and a resultant decrease in the percentage of pancreatic CSC, we evaluated the functional impact on CSC function by testing the effects of GSI in tumorsphere assays. Consistent with a dose dependent effect on Notch pathway inhibition, treatment of pancreatic cancer cells with GSI caused a dose dependent decrease in primary tumorsphere formation frequency (Figure 3A, p<.01 vs. control).

To determine if the observed inhibition of tumorsphere formation was preserved following transient exposure to Notch inhibition, treated tumorspheres were dissociated into single cells and re-cultured in normal sphere media without GSI. Notably, the effect on tumorsphere formation was preserved even after removal of the gamma secretase inhibitor, as evidenced by decreased generation of secondary tumorspheres (Figure 3A, p<.01 vs. control).

An effect of GSI on apoptosis could explain this reduction in tumorsphere formation. Indeed, we observed an increased rate of apoptosis based on PI and AnnexinV staining of cells from in tumorspheres treated with GSI compared to vehicle treated tumorspheres (Figure 3B, p<.05 8 μM vs. control, p<.01 16 μM vs. control). This result suggests that apoptosis contributes to decreased tumorsphere forming capability of the cells treated with GSI. We also performed cell cycle analysis in the absence and presence of GSI which revealed increased accumulation of cells in G1 with GSI compared to control (p<.01). Impaired cell cycle progression may therefore also contribute to reduction in tumorsphere formation with inhibition of Notch signaling pathway with GSI (Figure 3C).

In order to further validate this observed functional effect of in vitro Notch pathway inhibition, we studied whether pretreatment with GSI could inhibit tumor formation in vivo. In this assay, we pre-treated pancreatic cancer cells established as tumorspheres with GSI for 48 hours and then implanted viable, treated cells subcutaneously into NOD/SCID mice (5 mice per group). Cells that were untreated formed tumors 5 days earlier than those pretreated with GSI and grew significantly larger (p<.01) than those emanating from cells pre-treated with GSI (Figure 3D).

### Notch pathway inhibition using Hes1 shRNA

**Figure 4:**
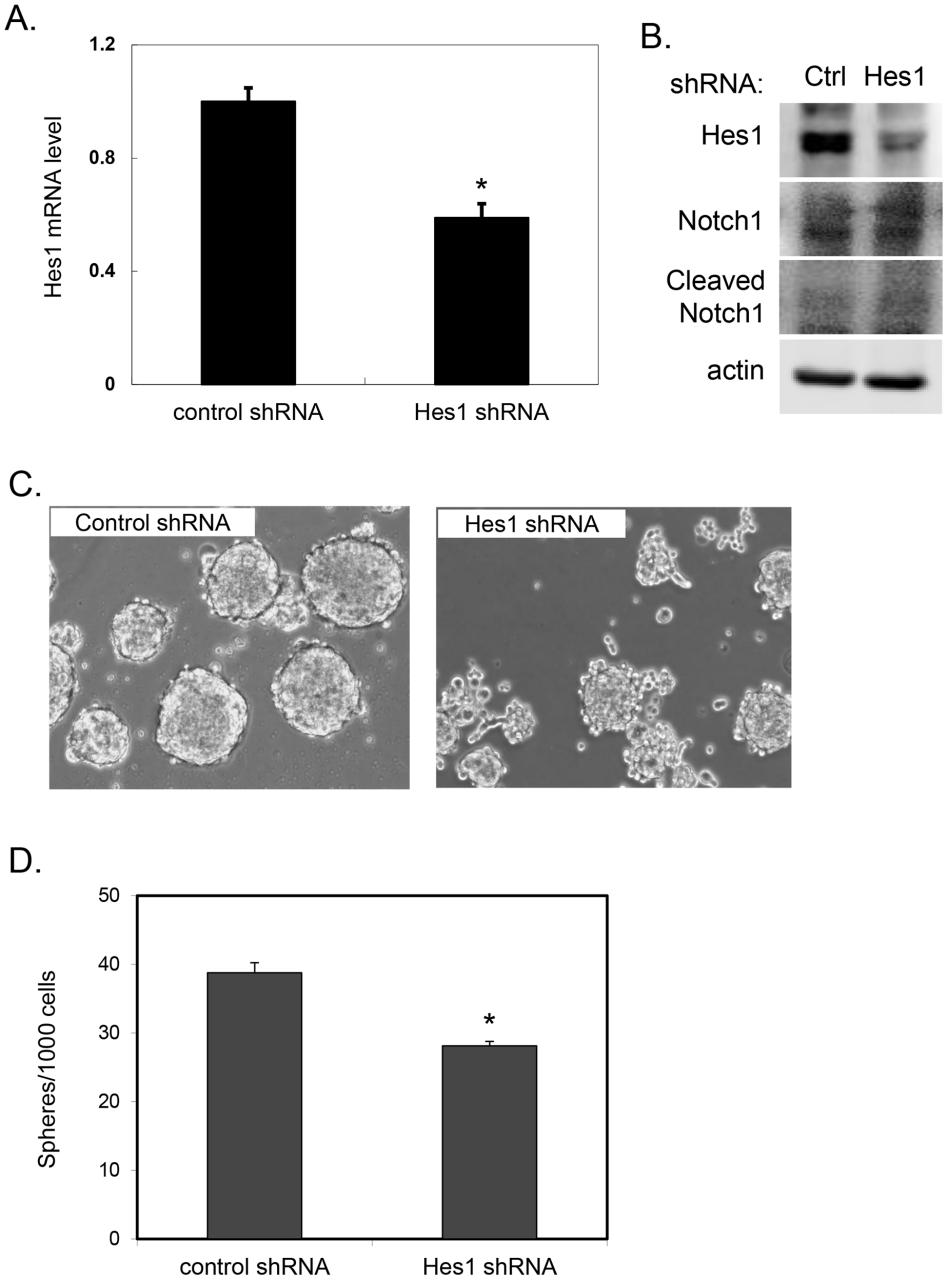
Hes1 shRNA treatment effect on pancreatic tumorspheres. **A**. Pancreatic cancer cells transduced with either control or Hes1 shRNA harvested after culturing for 4 days. Hes1 transcript levels quantitated by qRT-PCR, normalized to GAPDH (*p<.001 vs. control). **B**. Cell lysates were Western blotted for Hes1, Notch1, cleaved Notch1, or β-actin. **C**. Representative images of tumorspheres generated from pancreatic cancer cells transduced with either control scramble sequence or shRNA to Hes1 cultured for 5 days. **D**. Quantitation of number of tumorspheres generated per 1000 cells plated (*p<.001 vs. control).

As observed in Figure 1B, the mRNA level of Hes1, a direct target/effector of Notch-signaling, was upregulated in CSCs compared to non-CSCs in several primary xenografts. Upregulation of Hes1 was consistently observed across xenografts, in contrast to other Notch targets such as Hey1 and HeyL (data not shown). In order to assess whether the observed effects of GSI were specifically the result of inhibiting the Notch signaling pathway and not off-target effects, we used lentiviral constructs expressing control and Hes1 shRNA to specifically target Hes1. Knockdown of Hes1 by shRNA was confirmed at both the mRNA (p<.001 vs. control) and protein levels (Figure 4A,B) and did not affect upstream Notch1 cleavage (Figure 4B). Downregulation of Hes1 resulted in impaired tumorsphere formation (Figure 4C,D, p<.001 vs. control) providing further evidence that Notch pathway activation contributes to pancreatic CSC function.

### Ligand activation of Notch pathway promotes tumorsphere formation

**Figure 5:**
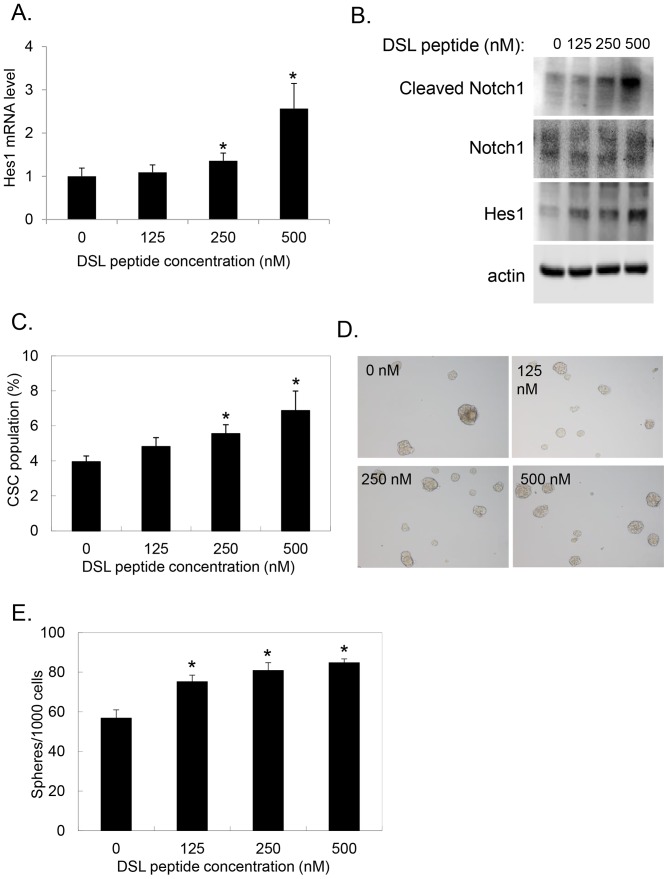
Notch pathway activation with DSL peptide stimulates tumorsphere formation and increased CSC population. **A**. Pancreatic cancer cell tumorspheres were cultured in sphere medium containing increasing concentration of DSL peptide as indicated for 5 days. mRNA levels of Hes1 from treated tumorspheres were analyzed by qRT-PCR and normalized to internal control GAPDH (*p<.001 vs. control). **B**. Tumorspheres cultured as in **A**. were Western blotted for cleaved-Notch1, Notch1, Hes1 or β-actin. **C**. Pancreatic cancer cells cultured in sphere medium containing increasing concentration of DSL analyzed for percentage of CSC by FACS analysis of cells stained with DAPI and antibodies to ESA, CD44, and CD24. Percentage of CSC represents ESA+/CD44+/CD24+ cells as a percentage of live DAPI-negative cells (*p<.02 vs. control). **D+E**. Tumorsphere formation assay performed on pancreatic cancer cells cultured in increasing concentration of DSL peptide. Representative images shown in **D**. with quantitation of number of tumorspheres per 1000 cells plated shown in **E**. (*p<.01 vs. control).

Activation of the Notch signaling pathway is dependent on receptor ligand activation with one of the Notch ligands. DSL is a synthesized peptide that mimics the conserved minimum sequence of the Notch receptor binding domain and may be used to activate the Notch signaling pathway [25], [26]. Hes1 expression increased in a dose dependent fashion at both the mRNA (p<.001 vs. control) and protein levels with exposure of pancreatic tumorspheres to DSL, confirming Notch signaling pathway activation (Figure 5A,B). Additionally, increasing levels of DSL led to increased cleavage of Notch1 ICD (Figure 5B). DSL exposure also led to an increase in the percentage of CD44+/CD24+/ESA+ pancreatic CSCs within tumorspheres (3.95%±0.33 vs. 6.87%±1.12 *p<.02 vs. control) (Figure 5C). This increase in CSCs induced by Notch pathway activation provides further evidence for an active role of Notch in pancreatic CSC maintenance. We tested the functional significance of this increase in number of CSCs by Notch pathway activation in the tumorsphere assay. Notably, Notch pathway activation by DSL exposure caused a dose dependent increase in tumorsphere formation (Figure 5D,E, p<.01 vs. control). These results confirm a functional contribution of Notch pathway activation to pancreatic CSC maintenance and function.

### GSI treatment inhibits tumor growth and decreases the CSC population in vivo

**Figure 6:**
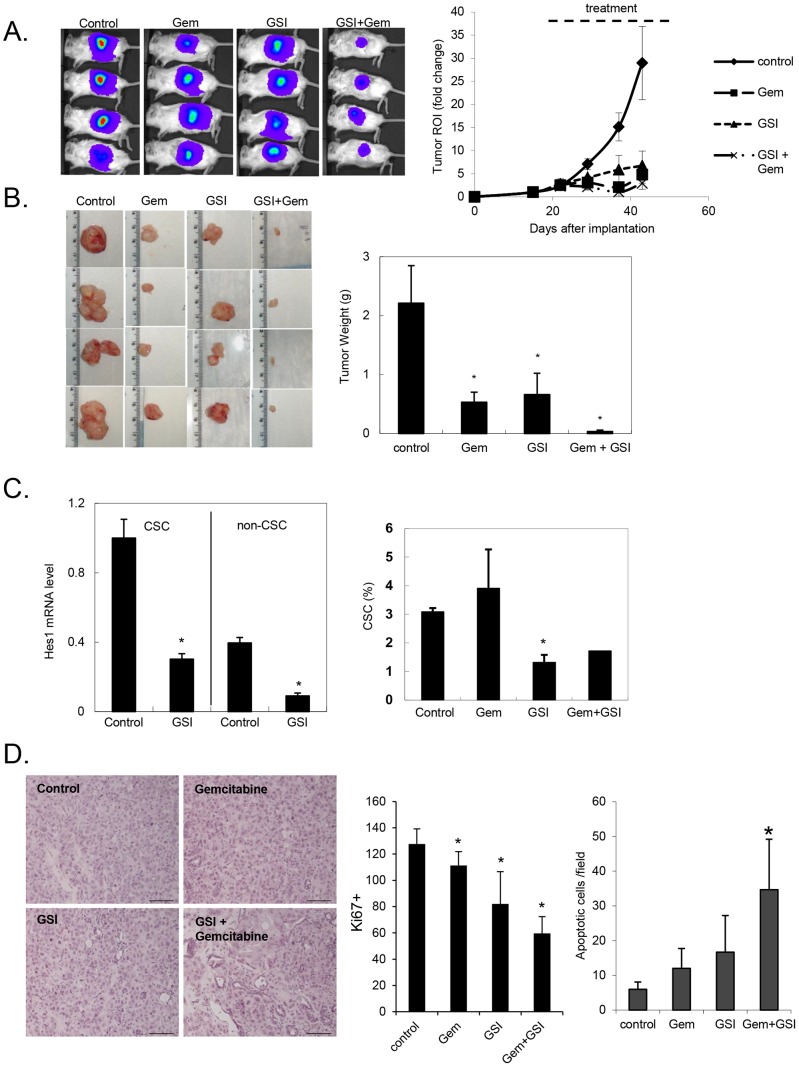
GSI treatment slowed tumor growth in an orthotopic model of primary pancreatic cancer. **A**. Representative images of tumors monitored using bioluminescent imaging. Fold change in bioluminescence of tumors over time from day of implantation. **B**. Images of tumors excised at the completion of 4 weeks of treatment for each treatment group. Final tumor weights (in grams) of the excised tumors (*p<.01 vs. control). **C**. (upper panel) mRNA harvested from tumors excised at completion of 4 weeks of treatment was analyzed by qRT-PCR for Hes1, shown normalized to GAPDH (*p<.02 vs. control). (Lower panel) Single cells were isolated from tumors excised at completion of treatment were analyzed for percentage of CSC by FACS analysis of cells stained with DAPI and antibodies to H2k, ESA, CD44, and CD24. Percentage of CSC represents ESA+/CD44+/CD24+ cells as a percentage of live DAPI-negative and human H2k-negative cells (*p<.01 vs. control). **D**. H+E stained sections from tumors treated with GSI, gemcitabine, or combination of GSI and gemcitabine. Proliferative index calculated as number of Ki67+ stained cells from formalin-fixed sections of tumor treated with drug. Graph representing mean number of Ki67+ cells per 40x field from 5 random sections from each of 3 tumors per treatment group (*p<.01 vs. control). TUNEL assay performed on sections of tumor treated *in vivo* and tabulated mean number of TUNEL+ apoptotic cells (*p<.001 vs. control).

Previous data suggests that pancreatic CSCs are more resistant to cytotoxic chemotherapy. Targeting the CSC compartment is thus a key strategy in any attempt to overcome this inherent limitation of currently available chemotherapy. The results described above provide compelling evidence for the importance of Notch signaling pathway activation in the maintenance and function of pancreatic CSCs. Notably, *ex vivo* treatment of pancreatic cancer cells with a Notch inhibitor reduced tumorigenic potential of the treated cells (Figure 3D). These results clearly support the Notch pathway as a potential therapeutic target in pancreatic cancer. To test the efficacy of targeting the Notch pathway *in vivo* in established tumors, we utilized an orthotopic pancreatic cancer model in NOD-SCID mice with low passage primary human pancreatic cancer xenografts. We used two of the primary pancreatic cancer xenografts profiled in Figure 1 to establish orthotopic tumors in the pancreas of mice. The cells were luciferase-labeled prior to implantation to allow *in vivo* bioluminescent monitoring of tumor growth. Once tumors were established and visible by bioluminescence (approximately 2 weeks), the mice were randomized to four treatment groups: vehicle control, GSI (30 mg/kg) alone, gemcitabine (50 mg/kg) alone, and a combination of GSI and gemcitabine. All mice tolerated the different treatments equally well without significant weight loss or noticeable change in physical activity. Mice treated with single agent GSI or gemcitabine demonstrated slower tumor growth during the 4-week treatment period compared to control animals (Figure 6A). Treatment with both GSI and gemcitabine further enhanced the inhibition, suggesting an additional benefit to the combination. At the completion of the 4-week treatment period, the mice were euthanized and the tumors were excised. The final tumor weights corresponded to the bioluminescence signal and confirmed tumor growth inhibition with treatment (Figure 6B, p<.01 vs. control).

To confirm that observed inhibition in tumor growth was due to Notch pathway inhibition, we performed assays on excised tumor tissue from each of the treatment groups. We first confirmed that oral delivery of GSI inhibited the Notch pathway activation in the tumor cells. Tumors were processed into single cell suspensions and subjected to FACS to separate ESA+/CD44+/CD24+ CSCs from non-CSCs. Transcript levels of Hes1 were reduced (p<.02) in both subpopulations as compared to control untreated tumors (Figure 6C). As was seen with *in vitro* treatment of pancreatic cancer cells with GSI (Figure 2C, D), *in vivo* inhibition of the Notch pathway reduced the number of CSCs within established orthotopic pancreas tumors (Figure 6C, p<.01 vs. control). These results support a direct therapeutic effect of Notch pathway inhibition in primary pancreatic cancers in an orthotopic primary tumor model.

Tumor histology was examined in all the treatment groups and was most notably altered with the combination of GSI and gemcitabine. Untreated tumors demonstrated densely packed cancer cells whereas combination treated tumors show evidence of necrosis and the tumor mass was less densely populated with cancer cells (Figure 6D). No significant difference was noted with either treatment alone. Our previously described cell cycle analysis following treatment with GSI in vitro suggested impaired cell cycle progression (Figure 3C). We therefore analyzed the proliferative index of tumors treated *in vivo* with GSI and confirmed a decrease in Ki67+ cells in tumors treated with GSI (p<.01 vs. control), an effect that was augmented with the addition of gemcitabine (Figure 6D and Figure S1). Having also shown by FACS analysis of *in vitro* treated cells that GSI increased apoptosis, we analyzed our *in vivo* treated tumors by TUNEL and further confirmed increased apoptosis in tumors treated with GSI (Figure 6D, p<.001 vs. control). The level of apoptosis in response to GSI was similar to that achieved with gemcitabine indicating that Notch signaling regulates both survival and proliferative pathway in PDAC. Tumors treated with combination of both GSI and gemcitabine had the highest amount of apoptosis suggesting enhanced efficacy of the combination. In addition to the direct effects seen with GSI alone, this latter result may reflect greater sensitivity to gemcitabine in a broader range of cancer cells due to inhibition of the Notch signaling pathway.

## Discussion

Pancreatic cancer is notoriously difficult to treat in part due to late detection, early spread, and resistance to therapy. Standard cytotoxic chemotherapy is provided with the intent to control disease but is well characterized to fall short of a clinically significant impact. While inherent heterogeneity of genetic changes within a tumor may explain differential sensitivity to chemotherapy, recent evidence supports the existence of a hierarchy of pancreatic cancer cells that contain a subpopulation of cells possessing stem cell characteristics like self-renewal. Self-renewal of normal stem cells has been shown to be dependent on several developmental signaling pathways including Wnt, Hedgehog and Notch [27]–[29]. These same developmental pathways have been notably reported to be upregulated in CSCs and to contribute to CSC function. We have previously shown that the Hedgehog ligand expression is upregulated in pancreatic CSCs over the already increased expression in bulk pancreatic cancer cells [10]. In the current study, we demonstrate that the Notch pathway is also further upregulated in the CSC compartment in comparison to bulk pancreatic cancer cells. Interestingly, although the Notch pathway has been shown to regulate the Hedgehog pathway through repression of GLI1 by Hes1 [30], we did not observe significant changes in Hedgehog signaling components when Notch signaling was inhibited (Figure S2A,B). The Notch pathway therefore represents a promising therapeutic target for pancreatic cancer.

Identification that a pathway is differentially up-regulated in cancer is clearly only the first step in determining its functional role in tumor growth. The concept of driver mutations versus passenger mutations reflects an observation that not all differential changes are necessarily sufficient for promoting tumor growth and metastatic behavior. The Notch pathway was recently identified as one of 12 core signaling pathways consistently altered in pancreatic cancer [31]. The role of the Notch pathway in development and its importance in normal stem cell maintenance points to potential importance in pancreatic CSC maintenance as well, and potential value of targeting this pathway in pancreatic CSCs has been proposed by some [32]. The results of the studies reported here validate the importance of Notch activation in pancreatic CSC maintenance. We utilized a novel inhibitor of gamma secretase to demonstrate that the Notch signaling pathway can be effectively targeted in pancreatic cancer cells. Inhibition of the Notch signaling pathway led to a decrease in CSC number and impaired CSC function. These effects were functionally relevant as the induced effects of Notch inhibition with a gamma secretase inhibitor decreased the ability of treated cancer cells to form tumors. Notably, we found that the effects of the gamma secretase inhibitor on CSC number (p<.05 vs. control) persisted weeks the drug was withdrawn (Figure S3), indicating a relatively stable response to pathway inhibition. The specificity of the observed effects due to gamma secretase inhibition was shown by attaining similar results in vitro through direct down-regulation of a Notch pathway target gene Hes1 with shRNA. Taken together, our results support that Notch pathway activation is necessary for pancreatic CSC maintenance and function. Notch receptor activation with the DSL synthetic ligand which directly activates the Notch signaling pathway, resulted in increased CSC number and function, as measured by tumorsphere formation.

Demonstrating that inhibiting the Notch pathway has functional consequences thus provides further evidence that this pathway is not only differentially expressed but plays a causative role in tumor growth. Ultimately, an observed pathway upregulation is only a useful therapeutic target if it can be shown that inhibiting it leads to tumor response. But chemotherapy alone has the potential to cause tumor response and yet clinical efficacy is again limited. In the CSC model, despite the sensitivity of bulk tumor cells to chemotherapy, CSCs are resistant and lead ultimately to the failure of cytotoxic chemotherapy. Targeting the Notch pathway leads to a tumor response as evidenced by the results of *in vivo* treatment of established orthotopic human pancreatic tumors with a gamma secretase inhibitor. These results are consistent with a recent study using a different gamma secretase inhibitor, PF-03084014, which also depleted pancreatic CSCs and enhanced the efficacy of gemcitabine *in vivo* [33]. These observations of synergistic effects of combining Notch pathway inhibition with chemotherapy provide insight into the potential of strategically targeting both CSCs and bulk tumor cells.

The potential clinical utility of such an approach carries much promise as one can clearly envision the clinical utility of targeting Notch pathway signaling for all stages of pancreatic cancer. In the setting of resectable disease, the observed high frequency of distant relapse following successful surgery suggests spread of cancer cells prior to surgery. CSCs have been shown to not only be resistant to therapy but also to have increased metastatic potential. By providing therapy to patients with resectable disease that targets CSCs through Notch inhibition, the subset of cancer cells responsible for recurrent disease and progression to an incurable state may be targeted. Prevention of pancreatic cancer metastasis by PF-03084014 gives credence to this idea [33]. In the advanced setting where disease is clinically evident in distant organs, systemic therapy is provided with palliative intent as it is well-established that chemotherapy can at best control disease for a period of time before eventual progression. CSCs may participate in this pattern of failure. Clinical benefit seen with bulk tumor reduction using standard chemotherapy could be greatly augmented by targeting CSCs to delay or even prevent this failure. Our study provides compelling preclinical evidence that such a strategy can be implemented in the treatment of pancreatic cancer and supports further exploration of targeting Notch signaling to target pancreatic CSCs in clinical trial design.

## Supporting Information

Figure S1 **Ki67 staining of GSI and gemcitabine treated xenografts.** Ki67 stained sections from tumors treated with GSI, gemcitabine, or combination of GSI and gemcitabine. Images were taken in 40x field and are representative of 5 random sections from each of 3 tumors per treatment group.
(TIF)

Figure S2 **Effects of HES1 knockdown and GSI on Hedgehog signaling.** **A**. Tumorsphere cells were transduced with either a non-targeting control shRNA or an shRNA targeting Hes1. Quantitative RT-PCR analysis of fold change in Hes1 and Hedgehog pathway components GLI1, GLI2, and PTCH1, normalized to a GAPDH control, are represented as vertical bars +/- SEM. Corresponding p-values between conditions are indicated. **B**. Primary PDAC cells were treated with RO4929097 (1 μM) or DMSO for 24 hours, after which mRNA was collected. Quantitative RT-PCR was performed and analyzed as in *A*. Corresponding p-values between conditions are indicated.
(TIF)

Figure S3 **Effects of GSI withdrawal on CSC subpopulation.** Primary tumor xenografts were established subcutaneously in NOD/SCID mice. Animals were treated daily with 30 mg/kg RO4929097 (5 mice) or vehicle (4 mice), 5 days on 2 days off, for 2 weeks. Tumor cells were harvested and expression of CD44, CD24 and ESA were analyzed by flow cytometry. An additional animal group (5 mice) was treated with 30 mg/kg RO4929097, 5 days on 2 days off, for 2 weeks, after which the treatment was ceased for 2 additional weeks. Tumor cells were harvested and analyzed by flow cytometry. Percent of cells co-expressing CD44, CD24 and ESA in each group is represented as vertical bars +/− SEM. Corresponding p-values between conditions are indicated.
(TIF)

Checklist S1 **ARRIVE Guidelines Checklist.**
(DOCX)
