# Supplementary figures and images for: The Notch Pathway Is Important in Maintaining the Cancer Stem Cell Population in Pancreatic Cancer
Source: PLoS One. 2014 Mar 19;9(3):e91983. doi: 10.1371/journal.pone.0091983 (PMC3960140; doi:10.1371/journal.pone.0091983)

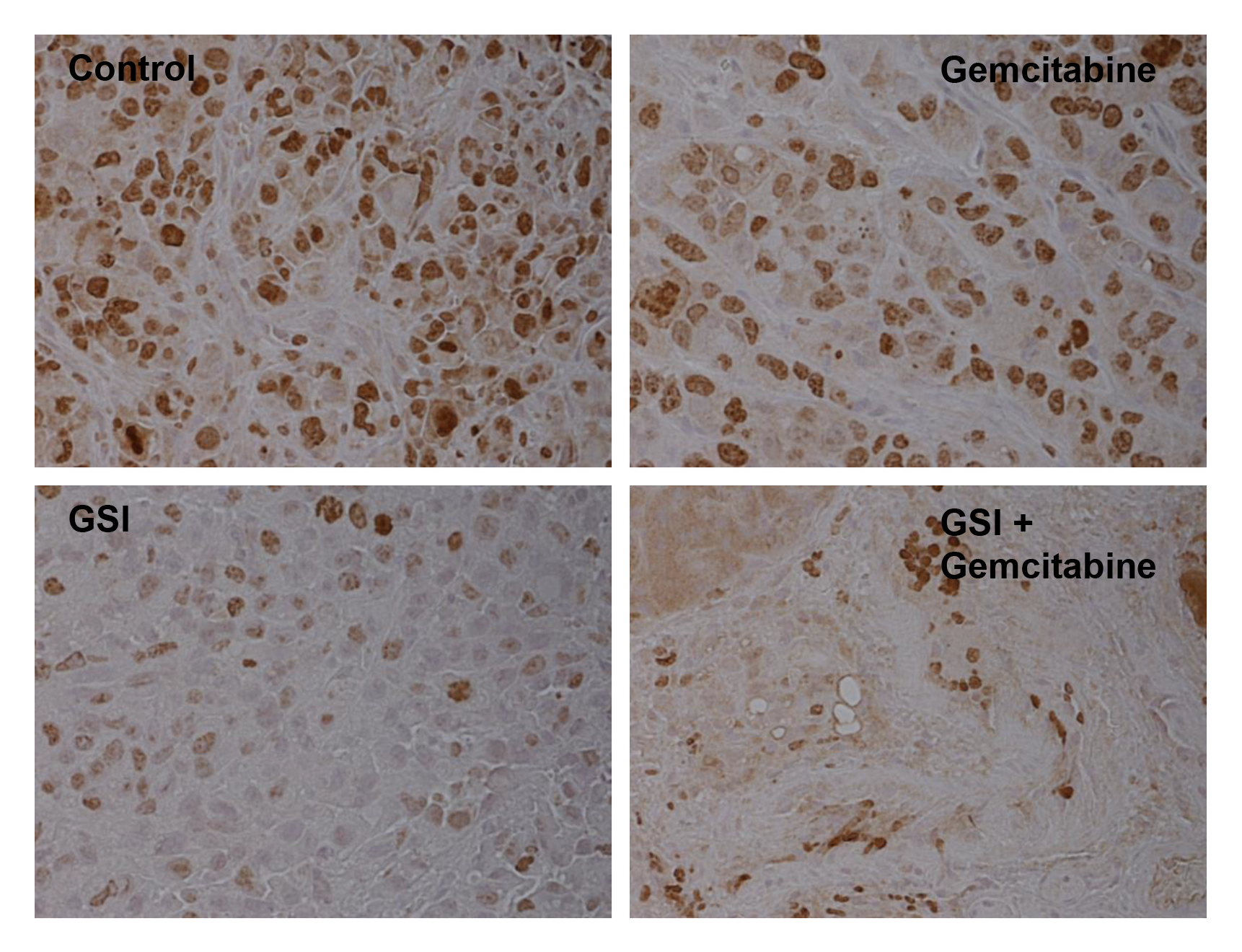

Supplement: Figure S1 — Ki67 staining of GSI and gemcitabine treated xenografts. Ki67 stained sections from tumors treated with GSI, gemcitabine, or combination of GSI and gemcitabine. Images were taken in 40x field and are representative of 5 random sections from each of 3 tumors per treatment group. (TIF) [file pone.0091983.s001.tif]

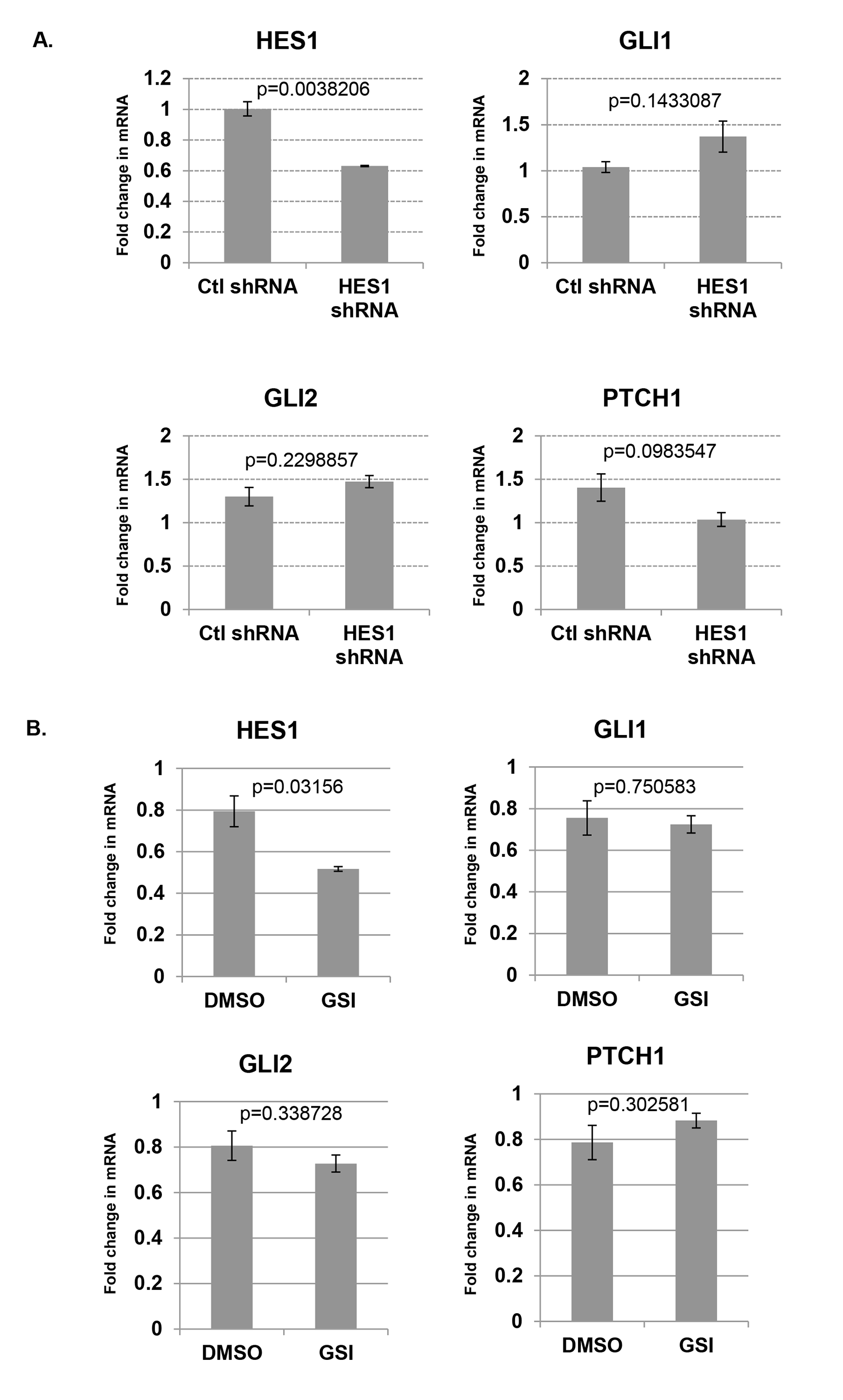

Supplement: Figure S2 — Effects of HES1 knockdown and GSI on Hedgehog signaling. A. Tumorsphere cells were transduced with either a non-targeting control shRNA or an shRNA targeting Hes1. Quantitative RT-PCR analysis of fold change in Hes1 and Hedgehog pathway components GLI1, GLI2, and PTCH1, normalized to a GAPDH control, are represented as vertical bars +/- SEM. Corresponding p-values between conditions are indicated. B. Primary PDAC cells were treated with RO4929097 (1 μM) or DMSO for 24 hours, after which mRNA was collected. Quantitative RT-PCR was performed and analyzed as in A. Corresponding p-values between conditions are indicated. (TIF) [file pone.0091983.s002.tif]

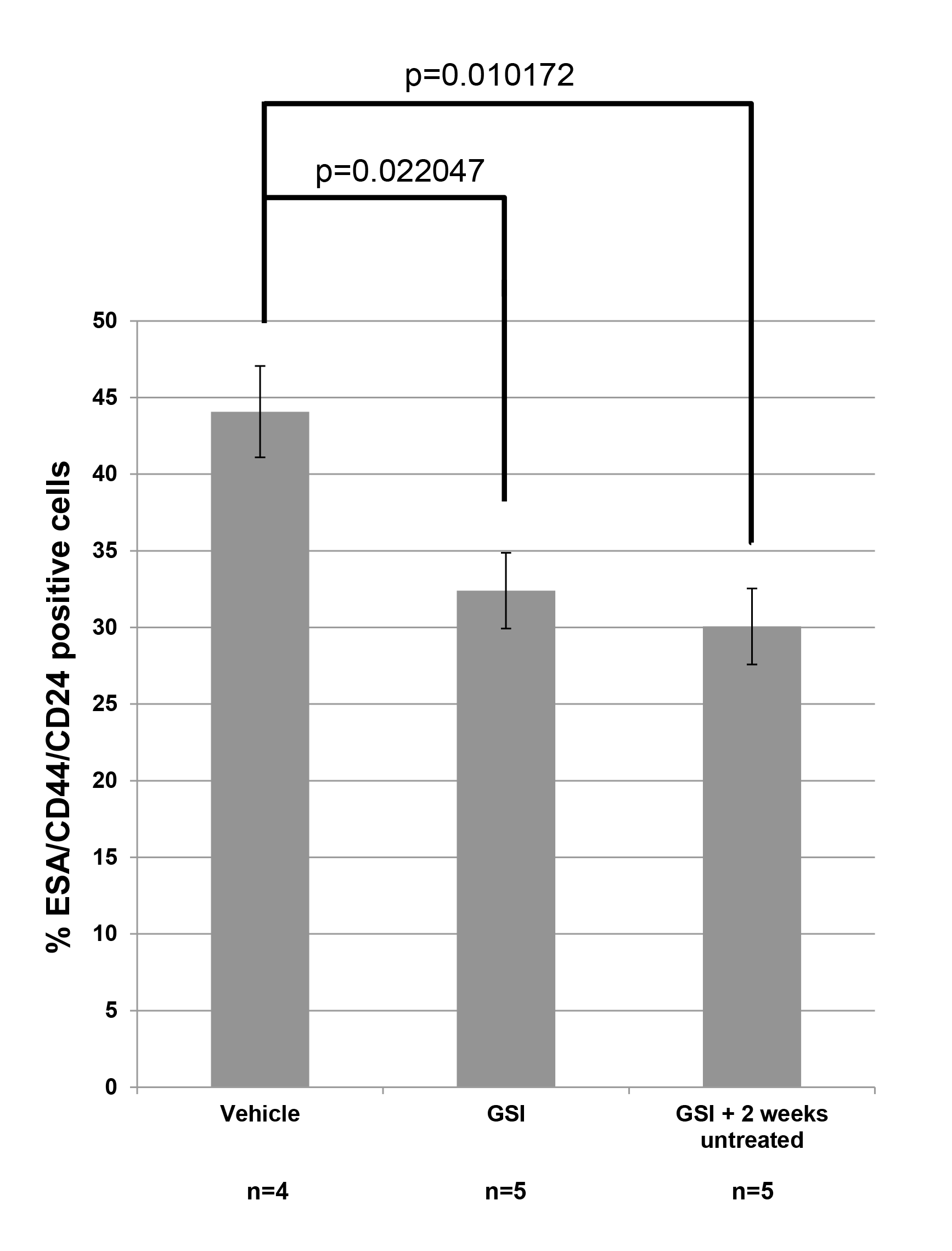

Supplement: Figure S3 — Effects of GSI withdrawal on CSC subpopulation. Primary tumor xenografts were established subcutaneously in NOD/SCID mice. Animals were treated daily with 30 mg/kg RO4929097 (5 mice) or vehicle (4 mice), 5 days on 2 days off, for 2 weeks. Tumor cells were harvested and expression of CD44, CD24 and ESA were analyzed by flow cytometry. An additional animal group (5 mice) was treated with 30 mg/kg RO4929097, 5 days on 2 days off, for 2 weeks, after which the treatment was ceased for 2 additional weeks. Tumor cells were harvested and analyzed by flow cytometry. Percent of cells co-expressing CD44, CD24 and ESA in each group is represented as vertical bars +/− SEM. Corresponding p-values between conditions are indicated. (TIF) [file pone.0091983.s003.tif]
