# Supplementary material for: The Notch Pathway Is Important in Maintaining the Cancer Stem Cell Population in Pancreatic Cancer
Source: PLoS One. 2014 Mar 19;9(3):e91983. doi: 10.1371/journal.pone.0091983 (PMC3960140; doi:10.1371/journal.pone.0091983)
Supplement: Checklist S1 — ARRIVE Guidelines Checklist. (DOCX) [file pone.0091983.s004.docx]

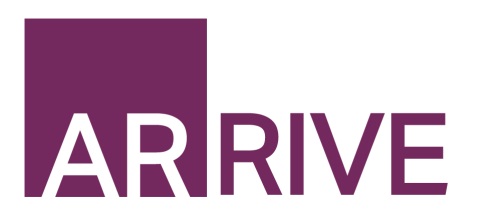


The ARRIVE Guidelines Checklist

Animal Research: Reporting In Vivo Experiments

Carol Kilkenny^1^, William J Browne^2^, Innes C Cuthill^3^, Michael Emerson^4^ and Douglas G Altman^5^

*^1^The National Centre for the Replacement, Refinement and Reduction of Animals in Research, London, UK, ^2^School of Veterinary Science, University of Bristol, Bristol, UK, ^3^School of Biological Sciences, University of Bristol, Bristol, UK, ^4^National Heart and Lung Institute, Imperial College London, UK, ^5^Centre for Statistics in Medicine, University of Oxford, Oxford, UK.*

|  | ITEM | RECOMMENDATION | Section/ Paragraph |
| --- | --- | --- | --- |
| Title | 1 | Provide as accurate and concise a description of the content of the article as possible. | The Notch Pathway is Important in Maintaining the Cancer Stem Cell Population in Pancreatic Cancer |
| Abstract | 2 | Provide an accurate summary of the background, research objectives, including details of the species or strain of animal used, key methods, principal findings and conclusions of the study. | Background: Pancreatic cancer stem cells (CSCs) represent a small subpopulation of pancreatic cancer cells that have the capacity to initiate and propagate tumor formation. However, the mechanisms by which pancreatic CSCs are maintained are not well understood or characterized. Methods: Expression of Notch receptors, ligands, and Notch signaling target genes was quantitated in the CSC and non-CSC populations from 8 primary human pancreatic xenografts. A gamma secretase inhibitor (GSI) that inhibits the Notch pathway and a shRNA targeting the Notch target gene Hes1 were used to assess the role of the Notch pathway in CSC population maintenance and pancreatic tumor growth. Results: Notch pathway components were found to be upregulated in pancreatic CSCs. Inhibition of the Notch pathway using either a gamma secretase inhibitor or Hes1 shRNA in pancreatic cancer cells reduced the percentage of CSCs and tumorsphere formation. Conversely, activation of the Notch pathway with an exogenous Notch peptide ligand increased the percentage of CSCs as well as tumorsphere formation. In vivo treatment of orthotopic pancreatic tumors in NOD/SCID mice with GSI blocked tumor growth and reduced the CSC population. Conclusion: The Notch signaling pathway is important in maintaining the pancreatic CSC population and is a potential therapeutic target in pancreatic cancer. |
| INTRODUCTION | | |  |
| Background | 3 | a. Include sufficient scientific background (including relevant references to previous work) to understand the motivation and context for the study, and explain the experimental approach and rationale.  b. Explain how and why the animal species and model being used can address the scientific objectives and, where appropriate, the study’s relevance to human biology. | Pancreatic cancer is a disease driven in part by cancer stem cells (CSCs), which drive tumor formation and are resistant to conventional chemotherapy. Notch-signaling is an important developmental signaling pathway in both normal stem cells, and in some cancers. We sought to determine whether Notch-signaling plays a role in pancreatic CSCs and whether targeting this pathway might be a potential therapeutic avenue. The animal models used in this study are immune-comprimised mice bearing primary human pancreatic cancer xenografts. The animals were treated with Notch-pathway inhibitors (GSIs) and conventional chemotherapy (gemcitabine) to determine whether Notch-pathway disruption alters tumor growth and biology, and as such, serves as a preclinical model for novel therapies targeting pancreatic cancer. |
| Objectives | 4 | Clearly describe the primary and any secondary objectives of the study, or specific hypotheses being tested. | The primary objective of these studies was to determine whether the Notch pathway is important for pancreatic cancer stem cells (CSCs), and whether targeting this pathways with gamma secretase inhibitors (GSIs) could block tumor growth, either alone or in combination with conventional chemotherapy. |
| METHODS | | |  |
| Ethical statement | 5 | Indicate the nature of the ethical review permissions, relevant licences (e.g. Animal [Scientific Procedures] Act 1986), and national or institutional guidelines for the care and use of animals, that cover the research. | The University of Michigan is in compliance with PHS policy on humane care and use of laboratory animals and is AAALAC accredited. Animal protocols were approved by University Committee for the Use and Care of Animals (UCUCA) at University of Michigan. The protocol number for these studies is PRO00005141. |
| Study design | 6 | For each experiment, give brief details of the study design including:  a. The number of experimental and control groups.  b. Any steps taken to minimise the effects of subjective bias when allocating animals to treatment (e.g. randomisation procedure) and when assessing results (e.g. if done, describe who was blinded and when).  c. The experimental unit (e.g. a single animal, group or cage of animals).  A time-line diagram or flow chart can be useful to illustrate how complex study designs were carried out. | For in vivo experiments in Figure 3, 5 control and 5 GSI-treated animals were used. For animal studies in Figure 6, 4 treatment groups (vehicle, GSI, gemcitabine, and GSI with gemcitabine) were used, each with 5 animals per group (20 animals total). This experiment was replicated with a different primary patient xenograft (20 additional mice). For persistence of GSI experiments (Supporting Figure 3), 15 animals were used (5 vehicle for 2 weeks, 5 GSI for 2 weeks, 5 GSI for 2 weeks with an additional 2 weeks without treatment). One vehicle animal did not form any tumor and was excluded from the end analysis. All treatment groups were seggretated by cage, with all treatments starting at the same time and the cages selected at random at the beginning of each experiment. In all in vivo experiments, the experimental unit was a tumor, one per animal, which was assessed for size and post-euthanasia for other properties (proliferation, apoptosis, Hes1 expression, CSC frequency). |
| Experimental procedures | 7 | For each experiment and each experimental group, including controls, provide precise details of all procedures carried out. For example:  a. How (e.g. drug formulation and dose, site and route of administration, anaesthesia and analgesia used [including monitoring], surgical procedure, method of euthanasia). Provide details of any specialist equipment used, including supplier(s).  b. When (e.g. time of day).  c. Where (e.g. home cage, laboratory, water maze).  d. Why (e.g. rationale for choice of specific anaesthetic, route of administration, drug dose used). | UCUCA guidelines for anesthesia, surgery , and euthanasiawere followed at all times. Gemcitabine was formulated in normal saline and administered once weekly by intraperitoneal injection (50mg/kg). Vehicle (proprietary from Roche) and GSI (RO4929097) was administered by oral gavage (30mg/kg), once daily, 5 days on, 2 days off. These doses and routes of administration were chosen based on doses/routes used in human subjects. Animals were treated in the morning in their home cages. For orthotopic implantation: mice were anesthetized with an intraperitoneal injection of 100 mg/kg ketamine and 5 mg/kg xylazine, and a small left subcostal incision was performed. 500,000 bulk tumor cells in a volume of 50 µl were injected into the tail of the pancreas using a 30-gauge needle. Buprenorphine was administered every 6 hours for the first 24 hours post-surgery to alleviate pain. For euthanasia: animals were euthanized by carbon dioxide asphyxiation followed by cervical dislocation to ensure death. |
| Experimental animals | 8 | a. Provide details of the animals used, including species, strain, sex, developmental stage (e.g. mean or median age plus age range) and weight (e.g. mean or median weight plus weight range).  b. Provide further relevant information such as the source of animals, international strain nomenclature, genetic modification status (e.g. knock-out or transgenic), genotype, health/immune status, drug or test naïve, previous procedures, etc. | NOD/SCID mice, originating from the Jackson laboratory, were used for this study. Both male and females, chosen at random at the start of experiments, were used. The average age of mice at the start of the experiment was 9 weeks, with an average weight of 21g for females and for 27g males. |

The ARRIVE guidelines. Originally published in *PLoS Biology*, June 2010^1^

| Housing and husbandry | 9 | | Provide details of:  a. Housing (type of facility e.g. specific pathogen free [SPF]; type of cage or housing; bedding material; number of cage companions; tank shape and material etc. for fish).  b. Husbandry conditions (e.g. breeding programme, light/dark cycle, temperature, quality of water etc for fish, type of food, access to food and water, environmental enrichment).  c. Welfare-related assessments and interventions that were carried out prior to, during, or after the experiment. | Animals were housed in a SPF facility, using, 5 mice per cage, in ventilated Allentown cages with sterile wood shavings as bedding. For breeding of NOD/SCID mice: A breeding colony has already been established using the ULAM Breeding Colony. In order to maintain genetic health and diversity, immunocompromised mice will need to be purchased and breed with existing animals. ULAM breeding colony managers have a set up a harem breeding scheme (each cage starting with 1 male and 2 females). The male is removed after the litters are born (or mother’s w/litters are separated depending on litter size). Acid Water may be administered to animals in the breeding colony, per vendor recommendations as a means to benefit the overall health of the animals. Animal welfare was assessed daily by the authors or by veterinary staff of the University of Michigan. All animals were either treated (where possible) or humanely euthanized at any sign of illness or stress. | |
| --- | --- | --- | --- | --- | --- |
| Sample size | 10 | | a. Specify the total number of animals used in each experiment, and the number of animals in each experimental group.  b. Explain how the number of animals was arrived at. Provide details of any sample size calculation used.  c. Indicate the number of independent replications of each experiment, if relevant. | For in vivo studies in Figure 3, 10 animals were used (5 vehicle, 5 GSI). For animal studies in Figure 6, 4 treatment groups (vehicle, GSI, gemcitabine, and GSI with gemcitabine) were used, each with 5 animals per group (20 animals total). This experiment was replicated with a different primary patient xenograft (20 additional mice). For persistence of GSI experiments (Supporting Figure 3), 15 animals were used (5 vehicle for 2 weeks, 5 GSI for 2 weeks, 5 GSI for 2 weeks with an additional 2 weeks without treatment). One vehicle animal did not form any tumor and was excluded from the end analysis. The number of 5 animals per group was arrived at by prior observations at this was the smallest number of animals that could be used to and still observe statistically significant changes. Additionally, all mice were housed at 5 mice per cage, and using this number of mice per group minimized disruption to neighboring mice and prevented the need of separating mice from their cage mates, minimizing stress on the animals. | |
| Allocating animals to experimental groups | 11 | | a. Give full details of how animals were allocated to experimental groups, including randomisation or matching if done.  b. Describe the order in which the animals in the different experimental groups were treated and assessed. | Equal numbers of animals were used for each experimental group within a given experiment. Each experimental group was confined to a separate cage. Cages were selected for a particular treatment at random at the start of treatment (all treatments were started at the same time). All groups were assessed at the same time (tumor monitoring/sacrifice/post-mortem analyses) except in experiments involving the persistance of the GSI's effects, where one treatment group was sacrificed two weeks after all other groups. All animals receiving GSI or vehicle control were treated on a once-daily, 5 days on, 2 days off schedule. All animals receiving gemcitabine were treated once a week. | |
| Experimental outcomes | 12 | | Clearly define the primary and secondary experimental outcomes assessed (e.g. cell death, molecular markers, behavioural changes). | Tumor growth, either measured by calipers (subcutaneous) or luminescence (orthotopic) was a primary experimental outcome of our in vivo studies. Secondary experimental outcomes included measurement of Hes1 mRNA levels by qRT-PCR, measurement of CSC frequency by flow cytometry, measurement of proliferation by Ki-67 staining, and measurement of apoptosis by TUNEL assay. All secondary outcomes were assayed using tumors taken from sacrificed mice at the end of tumor growth studies. | |
| Statistical methods | 13 | | a. Provide details of the statistical methods used for each analysis.  b. Specify the unit of analysis for each dataset (e.g. single animal, group of animals, single neuron).  c. Describe any methods used to assess whether the data met the assumptions of the statistical approach. | Statistically significant differences were determined by Student’s t test and Chi-squared analyses or the Mann-Whitney U test where appropriate, and defined as P < 0.05. The unit of analysis was a single tumor for in vivo and ex vivo analyses. | |
| RESULTS | | | |  | |
| Baseline data | 14 | | For each experimental group, report relevant characteristics and health status of animals (e.g. weight, microbiological status, and drug or test naïve) prior to treatment or testing. (This information can often be tabulated). | NOD/SCID mice, originating from the Jackson laboratory, were used for this study. Both male and females, chosen at random at the start of experiments, were used. The average age of mice at the start of the experiment was 9 weeks, with an average weight of 21g for females and for 26g males. All animals were assessed to be healthy and free of disease prior to tumor implantation. Tumors were allowed to establish until they were palpable or detectable by bioluminescence prior to drug treatments. | |
| Numbers analysed | 15 | | 1. Report the number of animals in each group included in each analysis. Report absolute numbers (e.g. 10/20, not 50%^2^).   b. If any animals or data were not included in the analysis, explain why. | For in vivo studies in Figure 3, 10 animals were used (5 vehicle, 5 GSI). For animal studies in Figure 6, 4 treatment groups (vehicle, GSI, gemcitabine, and GSI with gemcitabine) were used, each with 5 animals per group (20 animals total). This experiment was replicated with a different primary patient xenograft (20 additional mice). For persistence of GSI experiments (Supporting Figure 3), 15 animals were used (5 vehicle for 2 weeks, 5 GSI for 2 weeks, 5 GSI for 2 weeks with an additional 2 weeks without treatment). One vehicle animal did not form any tumor and was excluded from the end analysis. | |
| Outcomes and estimation | 16 | | Report the results for each analysis carried out, with a measure of precision (e.g. standard error or confidence interval). | In all experiments performed, GSI reduced the growth of tumors, as well as reduced proliferation and increased apoptosis. In experiments involving combination treatments with gemcitabine, GSI cooperated with gemcitabine and further increased cell death, reduced proliferation and reduced tumor growth. The measure of precision was standard error of the mean. | |
| Adverse events | 17 | | a. Give details of all important adverse events in each experimental group.  b. Describe any modifications to the experimental protocols made to reduce adverse events. | On adverse events were observed in this study. Dosing regimen for GSI was chosen to avoid adverse events observed by other groups (Tolcher et al, 2012, J Clin 6Oncol). | |
| DISCUSSION | | | |  | |
| Interpretation/ scientific implications | 18 | | a. Interpret the results, taking into account the study objectives and hypotheses, current theory and other relevant studies in the literature.  b. Comment on the study limitations including any potential sources of bias, any limitations of the animal model, and the imprecision associated with the results^2^.  c. Describe any implications of your experimental methods or findings for the replacement, refinement or reduction (the 3Rs) of the use of animals in research. | Our results indicate that inhibition of Notch-signaling with GSI reduces the number of CSCs in pancreatic cancer xenografts, and combined treatment with gemcitabine was more effective in eliminating tumor cells in vivo, supporting our original hypothesis. Limitations of this study are the number of primary samples available, therefore limiting our knowledge of how many patients might benefit from our proposed GSI/gemcitabine combination treatment. Additionally, differences in animal metabolism and the lack of a functional immune system may alter the results observed in xenograft studies versus human studies. The small number of animals used in any given experiment (5 animals per group) coupled with statistically significant results has implications for the 3R's by reducing the number of animals necessary for these types of experiments. | |
| Generalisability/ translation | 19 | | Comment on whether, and how, the findings of this study are likely to translate to other species or systems, including any relevance to human biology. | The tumors cells used in these studies are primary human samples, and all drugs used are either clinically approved for the treatment of cancer or in clinical trials for cancer. These animal studies serve as a preclinical model for combining treatment options for pancreatic cancer in humans, and the results justify the initiation of new clinical trials combining gemcitabine with GSIs. | |
| Funding | 20 | List all funding sources (including grant number) and the role of the funder(s) in the study. | | The Randy Pausch Family AACR-PANCAN Innovation Award provided the funding for these experiments. Other than providing funds, the funders did not oversee these studies. |  |


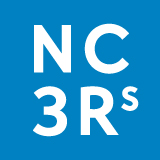


References:

1. Kilkenny C, Browne WJ, Cuthill IC, Emerson M, Altman DG (2010) Improving Bioscience Research Reporting: The ARRIVE Guidelines for Reporting Animal Research. *PLoS Biol* 8(6): e1000412. doi:10.1371/journal.pbio.1000412

2. Schulz KF, Altman DG, Moher D, the CONSORT Group (2010) CONSORT 2010 Statement: updated guidelines for reporting parallel group randomised trials. *BMJ* 340:c332.
